# The Hospital Nursing Supplement

**Published:** 1895-06-08

**Authors:** 


"The Hospital" Nursing Mirror.

BEING THE EXTRA NURSING SUPPLEMENT OF "THE HOSPITAL" NEWSPAPER.

[Contributions for this Supplement should be addressed to the Editor, THE HOSPITAL, 428, Strand, London, W.C., and should have the word "Nursing" plainly written in left-hand top corner of the envelope.]

### News from the Nursing World.

### THE HOSPITAL FOR SICK CHILDREN.

ON the right hand side of Powis Place stands the new block of buildings which is likely to prove a most valuable auxiliary to the Great Ormond Street Hospital for Children. Before occupation by patients the wards and nurses' quarters were thrown open for the inspection of visitors, and on May 31st and June 1st a large number of guests availed themselves of the opportunity offered them. The top floor will be used for measles, and below that diphtheria is to be nursed, the cots being intended for patients developing infectious diseases whilst already under treatment in the general wards. The walls are of glazed bricks, and the furniture is polished, and each set of wards is self-contained, having its own lavatory, bath-room, and kitchen. The nurses' rooms are bright-looking and suitably furnished, and their appearance forms an agreeable contrast to the inadequate provision made for the nursing staff in the block last opened at this hospital. The arrangements made for the reception of the visitors last week reflected great credit on the secretary, Mr. Adrian Hope, and the lady superintendent, Miss Smedley, by whom strangers were courteously welcomed and entertained at tea.

### "THE HOSPITAL" CONVALESCENT FUND.

"I AM sure you will be pleased to hear I am already feeling better for the sea air and good living," writes a nurse whom THE HOSPITAL Convalescent Fund has sent to recruit after a severe attack of influenza, which left her unfit for work. Her long illness and other circumstances rendered it impossible for her to take the holiday she so sorely needed without the assistance of the Fund established for nurses by nurses. "Please accept my warmest thanks for all the trouble taken on my account. I hope to return quite fit for work," is also written with grateful acknowledgment of help received from THE HOSPITAL Convalescent Fund. Instead of the "Nurses' Bed" being available at one Home only it has been found convenient to select several places situated in different parts of England, which often lessens the expenses of the journey, besides giving a certain amount of choice to the applicants. A nurse whose health requires a change of air, which her own unaided means do not allow her securing, should make written application to the Hon Secretary, the H.C.F., care of the Editor of THE HOSPITAL. It is desirable that she should give a short explanation of her requirements, and also the address of a doctor or matron to whom her circumstances are known. The Fund is designed to help nurses to regain their health and to fit them for resuming work, and subscribers are invited to keep the hon. secretaries informed of cases brought before their notice in which an over-tired or convalescent nurse could be permanently benefited by a visit to the sea. Subscriptions are invited from those who know the value of seasonable holidays. All donations addressed to the Hon. Treasurer or Hon. Secretaries of the H.C.F. will be promptly acknowledged and gladly received.

### FRIEDENHEIM.

THE "Home of Peace for the Dying," generally known as "Friedenheim," has issued an appeal for £2,000 to place the finances on a sound basis. The home is free from debt, and the management reflects great credit on the lady at the head of the establishment. It would be difficult to find a better ordered household, and the bright cleanliness observable in every department is quite a distinguishing characteristic of Friedenheim. The fine garden is a source of pleasure to those who can enter it, and in the balconies even the most feeble can often lie and watch the trees and flowers. The home is intended for those who are friendless, poor, and sick unto death. It is neither for chronic cases nor for the class of patients for whom poor law infirmaries exist. Friedenheim is a refuge for those to whom workhouse wards would add greatly to the bitterness of death. It is rather for poor clerks, governesses, small shopkeepers, and others who contribute according to their means, or if destitute are admitted free. The fine lofty rooms of this beautiful house form ideal wards, with cross currents of fresh air. Flowers are in profusion everywhere, and many comforts have been given, and much excellent furniture by various friends. "I never ask for things," said Miss Davidson, but the sight of her practical good work forms the best possible appeal for subscriptions. Anyone desiring to judge of the home and of its many advantages should take the train to Swiss Cottage and go and see Friedenheim. There is an adequate day and night nursing staff, and the neat dresses and caps and general trim appearance of the workers corresponds with the seemliness of their surroundings. There are forty beds for patients, including those in private rooms, for which payment is made. Cases of cancer and phthisis in the last sad stages are admitted, and there is no hard and fast religious test applied to those who enter. A trained sister does the dispensing and supervises the nursing, but Miss Davidson is her own housekeeper, and a visit to her home gives some insight into the admirable work which has been accomplished by the perseverance of one able gentlewoman.

### MATRONS OF COTTAGE HOSPITALS.

PRETTY, useful, and comfortable are many of the cottage hospitals dotted about the country, and in manufacturing and mining districts, and a nurse gains there such experience as few even large general hospitals could give her. Urgent illness and ghastly accidents have to be dealt with pending the arrival of the doctor, and, in carrying out his orders, a matron is practically "special nurse" to every serious case, in addition to housekeeping and generally supervising her little hospital. Her only helpers often consist in one probationer and a general servant, so that cooking invalid dishes must often come within the matron's duties. Cases of tracheotomy, strangulated hernia, double pneumonia, and compound fractures are amongst those by which a cottage hospital is liable to invasion at any time, and the better the nurse the more serious are the cases which doctor and neighbours contentedly place under her care. However excellent she may be as nurse and manager, the cottage hospital matron is but mortal, and she cannot work all day and be subject to calls during the night without suffering eventually from the want of sleep and the over-fatigue. How many ladies' committees or casual visitors realise that the health of a valuable worker is remorselessly undermined by the false economy of denying her competent temporary assistance? Cottage hospitals have charming wards and pretty accessories, but they are often deficient in accommodation for nurses; therefore a conscientious, unselfish matron will ruin her own health rather than demand an additional nurse, who must perforce occupy by day the tiny bed-room in which another sleeps at night.

### PRACTICAL ORDERS.

Excellent directions respecting the employment of the nurse engaged by the Birmingham Hospital Saturday Fund are issued in the current number of the journal of that association. The nurse's services are to be confined to the sick room, and she is only allowed to go to cases attended by a doctor, which shows that the subscribers properly discriminate between the duties of a nurse and a charwoman. They also note that "it is most important that the nurse should be summoned at the beginning of serious illness. Much ground is often lost owing to the doctor's instructions being neglected or badly carried out." Applications for the nurse's services are to be made on forms obtained from the offices of the Hospital Saturday Fund, 7a, Newhall Street, Birmingham.

### ECONOMY *VERSUS* EFFICIENCY.

The report of the committee appointed by the Slough Guardians to inquire into the nursing at the workhouse met with much adverse criticism at a recent Board meeting. The Guardians acknowledged the favour which skilled nursing enjoyed in adjacent unions, and the approval given to it by the Local Government Board; but, according to the report in the local press, they were far from unanimous in desiring to reform their own system. On economic grounds some of the Guardians desired that both structure and nursing should be left untouched. Even after attention had been called to the large number of inmates in the sick wards, and the class of cases there, the report of the committee was not adopted, notice being given that a proposal would be laid before the next meeting to appoint one nurse who could live outside the house. Efficiency is likely to be sacrificed to economy, unless the Guardians see the wisdom of adopting proved modern methods rather than those whose sole recommendation appears to lie in their having been endured for twenty-seven years.

### ENDANGERED LIVES.

Under the heading of "Serious Charges Against a Midwife," the *Sanitary Record* reports a somewhat surprising decision recently arrived at in the Sunderland Borough Police-court. It was proved by the health authorities that Jane Burlinson had attended, in the capacity of midwife, a Mrs. Gardner, who was certified in the course of a few days to be suffering from puerperal fever. Although the medical officer of health cautioned Jane Burlinson against going to other cases, she shortly attended a Mrs. Jopling, who developed the same fever on the day following the midwife's arrival. The medical officer said that he saw the latter in the clothes which she had worn whilst nursing Mrs. Burlinson. The sanitary inspector's evidence was to the same effect, and although no doubt was thrown on the truth of these serious accusations, the defendant was allowed to go free. She was merely cautioned not to attend any more cases for two months, "it being understood that if she broke her agreement she would be liable to be brought up again on the present charge." Sunderland seems likely to be regarded as a sanctuary in which ignorant and obstinate women can with impunity endanger the lives of their neighbours.

### A LEGACY FOR BUCKS.

One of the villages in Buckinghamshire, where Miss Nightingale's Health-Missioners carry on their useful work, is likely to be the chosen site of a cottage hospital. Amongst other handsome bequests to charities the late Dean of Ripon has left £1,000 to be invested towards the maintenance of a cottage hospital in either Middle, East, or Steeple Claydon, in compliance with the request of his late wife, and "in loving remembrance of Emily Verney."

### MENTAL AND BODILY AFFLICTION.

"Three months with hard labour" was the sentence passed at Scarborough Police-court the other day on Mary Ann Congdon for her assault on a private patient. The relations of the latter engaged two "professional nurses" to take care of her pending her removal to an asylum, and the unfortunate lady was beaten and ill-used by Mary Ann Congdon, who was under the influence of drink. The other "nurse" witnessed the assault, but, according to the report in the local press, she did not even summon help, and when questioned on this point in the police-court, said she "felt paralysed with terror." No evidence seems to have been given in explanation of the supply of champagne and brandy in the patient's room, of which Congdon is said to have freely partaken; nor does anyone appear to have come to the rescue of the poor victim to such cruel treatment at the hands of one woman in the presence of another.

### PROGRESS IN ROME.

The institution of a training school in Rome for teaching Italian women to nurse the sick poor in their own homes has the support of Professors Rossini, Mazzoni, and Tosti. Hitherto no district nurses have been supplied to the Italians, and this new plan is widely approved and encouraged. It is proposed that two years' training should be given in one of the larger hospitals, where instruction could be given by the medical staff and the sisters. It will, of course, be essential that the latter be themselves fully-trained nurses.

### SHORT ITEMS.

"Nursing Notes" for June contains an article on "Women as Dispensers," besides much interesting information regarding the Midwives Registration Bill. —Tredegar House, which has been established in connection with the London Hospital Training School for Nurses, will receive the first twenty pupil probationers on June 29th.

### Elementary Anatomy and Surgery for Nurses.

By W. McAdam Eccles, M.B., M.S., F.R.C.S., Lecturer to Nurses, West London Hospital, &c.

### XX.—HÆMORRHAGE FROM PARTICULAR REGIONS.—ANEURISM.—NÆVUS.

*Hæmorrhage from the mouth and pharynx.*—Troublesome bleeding may sometimes occur from the socket from which a tooth has been extracted. It can usually be readily stopped by rinsing the mouth out with ice-cold water, but in certain cases requires that the cavity be plugged with some cotton wool which has been steeped in a solution of the perchloride of iron. After operations on the tongue hæmorrhage may supervene, and, if excessive, a nurse should remember that she can always temporarily arrest it by hooking a couple of fingers round the base or stump of the tongue and drawing it forward against the lower jaw. In this manner the lingual artery is firmly compressed. Sometimes there is a good deal of oozing after excision of the tonsils, or removal of growths from the naso-pharynx. Gargling with ice-cold water is efficacious if the bleeding is excessive. It is well to know that the blood in these cases is frequently swallowed, and may afterwards be vomited.

*Hæmorrhage from the nose* (*Epistaxis*).—This may sometimes be so large in amount as to be serious. The chief causes of epistaxis are: (1) Congestion of the mucous membrane by no means infrequent in children about 14 years of age; (2) injury to the nose; (3) polypus of the nose; (4) in plethoric persons; (5) in the old with diseased vessels; (6) in some of the specific fevers, and in "bleeders." The hæmorrhage generally occurs from one nostril, and the blood not infrequently trickles down the back of the throat. The usual methods of treatment are placing the patient in a semi-recumbent position with the arms raised and allowing him to suck ice, or injecting cold or decidedly hot water into the affected nostril. In cases where such treatment is unavailing it may be necesssary for the surgeon to plug the nares.

*Hæmorrhage from the palm of the hand* is sometimes very troublesome. It is here difficult to ligature vessels without doing considerable damage to the parts, and therefore a graduated compress is generally employed. This should be made of gauze and built up into the shape of an inverted pyramid. A broad splint for the hand, and bandages for the fingers and forearm should be prepared. It is a good rule in all cases of arterial hæmorrhage to endeavour to keep the patient quiet in a recumbent position, and to endeavour to apply pressure to the main artery of the part on the heart side of the source of the hæmorrhage.

### Aneurism.

An aneurism is a tumour connected with an artery, often by a comparatively small orifice, and into which blood is pumped by every beat of the heart. It is generally the outcome of a part of the wall of a vessel giving way, and the force of the blood stream causing more and more dilatation of the sac. The aneurism will have a great tendency to compress important organs, and to rupture and lead to fatal hæmorrhage. Aneurisms may occur on any artery, but those within the great cavities of the body—the thorax and the abdomen—are styled internal, and can only be dealt with in the majority of cases by medical means, and those which are found in one of the limbs or the head and neck are termed external, and in many instances can be satisfactorily cured by surgical treatment. The latter method only will be noticed here. The aim of such is to limit the circulation in the artery which supplies blood to the tumour, and thus to produce a likelihood of the blood clotting in the sac. This may be brought about by two ways. The first is to apply pressure, if possible, to the vessel of the proximal side of the tumour, either by especially constructed instruments, or by the fingers of trained assistants. The second is to tie the vessel, again on the heart side of the swelling. This latter proceeding must necessarily gravely affect the circulation through the part and thus its vitality, and it becomes of the utmost importance that a nurse should understand the after treatment of such a case. The whole limb, if that be the area affected, should be thoroughly protected by being wrapped up in cotton wool, and warmth maintained by hot bottles being placed beneath the bed clothes, but nowhere in contact with the limb. The patient must be kept absolutely quiet, and every means taken to prevent gangrene, by raising the limb and protecting it from cold. In some cases secondary hæmorrhage may occur at the seat of the ligature; pressure over the site of the bleeding will control it temporarily while assistance from the surgeon is sent for.

### Nævi.

Nævi, which are very common, are of two varieties, the capillary and the venous. Capillary nævi consists of masses of dilated, anastomosing capillaries, forming tumours beneath the skin or mucous membrane. They are most commonly situated on the head, face, neck, or trunk, and are usually present at birth, but may grow in size afterwards. The blood in these small vessels can be pressed out temporarily, but only to return as soon as the pressure is removed. They seldom or never give rise to any serious hæmorrhage, unless accidentally wounded. The treatment usually adopted is to destroy them with a caustic. Venous nævi, on the other hand, are made up of spaces resembling dilated veins, and filled with blood of a dark venous colour. They are again congenital, and commonly found in connection with the skin or mucous membranes. This variety is a more serious affection than the capillary form, and may sometimes lead to bleeding, which is not always easy of control. They are best treated by excision or by electrolysis, which latter method consists in the insertion of needles into the tumour, which are connected with the poles of a battery, and thus passing a weak constant current through, causing decomposition and coagulation of the blood in the spaces.

### The Society of Trained Masseuses.

The following candidates passed the last examination held by the Society of Trained Masseuses: Misses Winifred Allott, Gertrude Gainsford, Ethel Hailey, Marion Ludbrook, Julia Newlyn, and Mrs. Addie Vane-Thomas. The next examination will be held in July, and particulars can be obtained of Mrs. Arthur, Hon. Secretary, 12, Buckingham Street, Strand.

### Appointments.

[It is requested that successful candidates will send a copy of their applications and testimonials, with date of election, to The Editor, The Lodge, Porchester Square, W.]

Crewe Memorial Cottage Hospital.—Miss Janet Rodgers has been appointed Matron at this hospital. She was trained and afterwards held the post of night sister at the London Hospital. Miss Rodgers afterwards had some experience in private nursing and in taking holiday duty, and was then made matron of the Hinckley Cottage Hospital. Her work there has been excellent, and she has been selected out of 92 candidates for the appointment at Crewe. Miss Rodgers will take the good wishes of many friends with her to her new work in which we wish her every success.

### Boston Training School for Nurses.

(Communicated.)

English nurses are sometimes interested in comparing the particulars of their training and work with that of their sisters in the profession on the other side of the Atlantic. A short account of the Boston Training School for Nurses (attached to the Massachusetts General Hospital, although a separate institution), which holds a high place among nursing schools in the United States, may therefore not be out of place in the columns of The Hospital.

The rules and regulations for would-be candidates are much the same in American training schools as in similar institutions in England. The age considered most desirable at the Boston Training School is from twenty-five to thirty-five. Candidates are required to refer to some responsible person as to character, &c., and must be in good health. After satisfying the resident physician of the hospital and the superintendent of the school on all such points, they are admitted for one month on probation, during which time they are boarded and lodged at the expense of the hospital, but receive no salary. Whether at the end of the trial month they are retained for the course of two years' training or not rests with the resident physician and the lady superintendent and directors of the school, and these authorities can also dismiss them at any time in case of misconduct or inefficiency. They are required, if accepted as pupils, to sign a written agreement for a two years' term of service, including the trial month, and undertaking to conform to the rules of the school.

The course of instruction is thorough, and quarterly examinations are held by a board of medical examiners. The instruction is principally supplied by the superintendent and by the head nurses. The remuneration offered to probationers is ten dollars a month (after the first) during the first year, and fourteen during the second for personal expenses; it being considered that the training they are receiving is a full equivalent for their services. Laundry expenses are borne by the hospital. At the end of the two years' course a diploma or certificate is given as to length of training, character, and proficiency, and a badge is provided by the training school, to which graduates are entitled on the payment of seven dollars.

Those nurses who have a special desire to go in for a study of nervous and mental diseases are privileged, upon certain conditions, to enter upon a year's training at the McLean Hospital Training School, at the termination of which they will, if they have there satisfied the authorities, receive a certificate from that school in addition to the one from the Boston School.

The past year, 1894, may be chronicled as one of very successful work, the pupils, both in numbers and character-keeping up a high standard. Funds are, however, much needed, if the work and usefulness of the institution are not to be curtailed. The training school, though, as already mentioned, it is attached to the Massachusetts General Hospital, is not a corporate part of that institution, and has to maintain itself and to meet its necessarily heavy current expenses from its own funds.

A development of the past year has been in the establishment of the cooking school on a new basis. Until 1894 this instruction was given outside the hospital, but the efforts of the directors have, with the kindly co-operation of the hospital trustees, resulted in the classes being now held in the hospital kitchen, under the direction of Miss Boland, whose name is sufficient guarantee for the excellence of the teaching under this head.

Some special instruction in the autopsy room has been supplied during the past year by one of the physicians to the hospital, which has been of very considerable value to the nurses, and much appreciated.

Altogether it may be taken that the system pursued at the Boston Training School, under the superintendence of Miss Maria B. Brown, is thorough and complete, and those who have passed successfully through the prescribed training should be well qualified to take their places in that great profession which has for its object the care of the sick and suffering.

### The Indian Hospital, British Columbia.

By An English Nurse.

Our "Indian Hospital" in British Columbia was consecrated and opened a year ago by our late Bishop, through whose untiring efforts the Government was persuaded of the necessity for its establishment. A small grant of $500 was eventually made, and to this sum the Bishop personally collected an addition of upwards of $1,000. With these funds in hand the construction of a neat little hospital, overlooking the rivers Fraser and Thompson, was commenced at Lytton.

The Indians are superstitious about leaving their own families when sickness overtakes them, and until a few years ago they were exclusively tended by their "medicine men," and had a great dread of real physicians. This is only one of many prejudices that missionaries have had to overcome. It reflects great credit on their teaching that now eight Indians out of ten will send to missionaries to ask for a white doctor to be sent to them, and will take their medicines and follow their directions.

About twenty-eight patients have been admitted to the hospital since the late Bishop Sillitoe gave the work into the hands of Sister Frances, of St. Luke's Home, Vancouver—the only stipulation made by him being that no debts should be incurred. At times both a matron and nurse have been at work, and, until lately, no money from outside the diocese has been received, the hospital being kept up by contributions. At present there are three inmates—two girls and a little boy. The latter, suffering from spinal complaint, occupies a cot which, with all equipments, was presented to the institution last summer by Messrs. Gale Bros., Waterville, Ontario.

Miss Berkeley, an English lady, is in charge and is kept busy, having many more out-patients than indoor ones. To the former she carries daily medicines and nourishment as required. Over a hundred Indians came in the past year for medicines, and more than half the number were visited by Miss Berkeley.

As we have just received our first quarterly cheque from a Government grant of $400 (for one year) we are in hopes of being able to offer part of that sum to a medical man, who we shall ask to reside at the hospital. We have been assured that if we advanced some money for this purpose a similar sum might be given in England, and, of course, a doctor is sadly wanted. Until we have a resident on or near "the reserve" we cannot expect great things of the Indian Hospital work. Sister Frances came to us in order to assist the Rev. Mr. Edwardes in getting up a Christmas tree for the native children, and, of course, they had never seen one before. Tea and cakes formed part of the entertainment, and every child on the Rancherie and the old people were invited. We look forward with great interest to the arrival of The Hospital, and we shall most eagerly watch if Lytton is named in its columns. In our tiny mountain home we were in no way behind our English sisters in endeavouring that Christmas should be observed in correct home fashion.

### Everybody's Opinion.

[Correspondence on all subjects is invited, but we cannot in any way be responsible for the opinions expressed by our correspondents. No communications can be entertained if the name and address of the correspondent is not given, or unless one side of the paper only be written on.]

### PRIVATE NURSING.

"DEMOCRAT" writes: "Sister Lucie" and "M. G." seem to be the only ones who have treated this subject fairly in the columns of THE HOSPITAL, and on the broad lines it deserves. Perhaps they have had longer experience and possess greater knowledge of the world than some of the other correspondents. Nobody but a private nurse can know of the "exceptions" in this line of life, which must be met as they come, and they are always coming! Perhaps I take an exaggerated view of nursing. I have always looked on it as the highest calling a woman can follow. "M. N." (April 20th) says she "finds no trouble in getting work in good county families at six guineas per month." Is it from associating so much with this class that she is led to think that all the rest of the world "look down" on nurses who can be useful in the sick-room and out of it? "Nurses do not go through a long period of training merely to fit them for looking after chronic cases or housework." May I ask if chronic cases are not to be numbered with the sick? Since it appears necessary to enlighten the public, I think we ought to thank the Editor of THE HOSPITAL for saying no nurse should be asked to take her meals with the servants. Infection brings exceptions of its own. But "M. G." is quite right; people generally show discernment. There is the retail tradesman who thinks much of his respectable position, and from whom I have received a higher fee than "M. R." gets from her "county families." An often forgotten yet numerous class in London consists of men in chambers or lodgings. Who shall say how much a nurse shall do there? The rooms badly kept, nobody else on the spot looking after the patient's interests. In a very large household a request may be made that the nurse shall take her meals in the steward's or housekeeper's room. Who will be found with sufficient courage to offend these mighty folks? And to these circumstances and many more a nurse must adapt herself. If she has self-respect none will "look down" on her except the narrow-minded and vulgar.

THE correspondent who wrote from Rotherhithe and did not sign her name cannot have her communication inserted, unless she sends both that and her address.

### Royal National Pension Fund for Nurses.

### PRESENTATION TO NURSES AT MARLBOROUGH HOUSE.

HER ROYAL HIGHNESS THE PRINCESS OF WALES, as President of the Pension Fund, has graciously intimated her wish to receive the third and fourth thousand nurses at Marlborough House on a day at the end of July. H.R.H. the Princess of Wales will present each nurse with a certificate which has been specially designed by Mr. Arthur Hacker, A.R.A., to whom the Council are greatly indebted for his kindness and generosity in the matter. "Our Princess" is unable to fix the precise date, but this intimation is given to enable all nurses included in the Third and Fourth Thousand to make arrangements for attending this interesting ceremony. We are requested to ask nurses not to apply to the Secretary of the Fund, as he is already preparing a circular-letter containing all needful particulars, which will be sent to them in due course.

### Notes and Queries.

The contents of the Editor's Letter-box have now reached such unwieldy proportions that it has become necessary to establish a hard and fast rule regarding Answers to Correspondents. In future, all questions requiring replies will continue to be answered in this column without any fee. If an answer is required by letter, a fee of half-a-crown must be enclosed with the note containing the enquiry. We are always pleased to help our numerous correspondents to the fullest extent, and we can trust them to sympathise in the overwhelming amount of writing which makes the new rules a necessity. Every communication must be accompanied by the writer's name and address, otherwise it will receive no attention.

### Queries.

(150) *Monthly Nurse.*—I shall be grateful for advice regarding any institution where a nur e who has received only monthly and midwifery training could be engaged for private work?—*Nurse G.*

(151) *Training.*—Where can a year's general training in nursing be obtained?—*Certified M. N.*

(152) *A Trained Nurse.*—Please tell me whether two years in a special private hospital can be reckoned as part of the three years' course of training now demanded of nurses.—*Probationer.*

(153) *Abroad.*—I have had nine years' experience as nurse and sister in a large northern hospital, and I shall be glad if you will write me full particulars of the best way to get a matron's post abroad.—*Nurse L.*

(154) *Riviera.*—Can you give me the address of institutions which send out nurses in the winter to the Riviera?—*S. Gamp.*

(155) *Times off Duty.*—Do you think it unreasonable for nurses and sisters to ask the matron to fix their monthly days off duty a little while beforehand? If this is done say two days in advance, we have the chance of letting our friends know, and so avoiding loss of time and disappointment on a day eagerly anticipated by most probationers. It is not unusual in the institution we are in for the leave granted beforehand to be cancelled at the last moment, which we find very trying.—*Fairplay.*

(156) *Recreation.*—Will you be so good as to give your opinion on the hours of work in an institution in which I have been working. The nurses are on from seven a.m. to half-past eight p.m., with two hours off either from ten to twelve, or from half-past two to half-past four. Every other Sunday we are off from two to nine, and on the alternate Sundays have two hours for morning service in the chapel. We have extra time off for meals, but when our turn on week-days is from half-past two to half-past four tea is included. We have to put our rooms in order during our morning two hours off duty.—*Fairplay.*

(157) *Benevolence.*—Can you give me the address of the Governesses' Fund.—*H. D. R.*

### Answers.

(150) *Monthly Nurse* (*Nurse G.*)—If you are duly certificated you had better consult the hon. secretary of the Midwives' Institute. You can either call or write, sending a stamped envelope for reply, to Mrs. Nichol, 12, Buckingham Street, Strand.

(151) *Training* (*Certified M.N.*)—As a paying probationer at the London Hospital, at Guy's, the Royal Free, the Middlesex Hospital, St Mary's, and others in the metropolis and provinces of which you will find the names in "Burdett's Hospital Annual" and "How to Become a Nurse." Both books are published by the Scientific Press, 428, Strand. We do not advise you to go to any hospital or infirmary with less than a hundred beds.

(152) *A Trained Nurse.*—The experience in a good private hospital may be a valuable addition to general hospital training, but it cannot take the place of it. We advise two or three years' training (under a trained matron) in any infirmary or general hospital, supplemented if desired by fever or other special work. We are aware that many probationers are induced to enter private hospitals and homes thinking they will get trained. As a rule girls thus employed are engaged for economical reasons as substitutes for the more expensive experienced nurses. We frequently hear of probationers leaving these homes and small hopitals after a year or two of hard work to begin from the beginning again in some recognised training school for nurses, that they may gain the only kind of certificate likely to serve them in the future.

(153) *Abroad* (*Nurse L.*)—We cannot possibly send letters in answer to all the questions sent to us. If you read the heading to our Queries you will understand this. You should answer advertisements or advertise in our columns.

(154) *Riviera* (*S. Gamp*).—You will find them in "Burdett's Hospital Annual." It is usual for the managers to advertise later in the year when they know how many former nurses are going out again, and therefore how many vacancies may require filling.

(155) *Times off Duty* (*Fairplay*).—Your demand is quite reasonable, and matrons who are good managers generally plan all the "times off" for their nursing staff some days in advance. You must, however, see that emergencies constantly arise in hospital life, when individuals must make sacrifices for the good of the general community. Although we are strongly opposed to regular holidays being curtailed or altered, we feel sure that no nurse worthy of the name would make a grievance of temporary personal inconvenience. Matrons who have the best interests of nurses at heart are unlikely capriciously to cancel the leave they have already sanctioned.

(156) *Recreation* (*Fairplay*).—Your hours seem to us fairly satisfactory, but it would be better if you all had half an hour in the morning for dressing and putting your rooms neat, in addition to the two hours recreation. We see no hardship in tea being included in your afternoon "time off," as that meal is restful and refreshing, and you probably return to your wards quite fit for the evening work. The Sunday leave is very satisfactory, and is no doubt highly appreciated. Your hours compare favourably, on the whole, with other institutions. A few of the best hospitals may be better, but the majority are less liberal. To those who took on nursing as their life-work, holidays and "times off" are regarded as means whereby mental and physical powers are cultivated and conserved. When a woman makes recreation hours her first consideration and lets duty take the second place she had better give up hospital nursing altogether.

(157) *Benevolence* (*H. D. R.*).—The Governesses' Benevolent Institution, 32, Sackville Street, W.

### Nursing in Military Hospitals.

### TIME-EXPIRED MEN.

Gosport is a sleepy town, and seems a small one, but it really straggles on far past the "New Barracks" for an infantry regiment. These barracks, report says, were intended for Hong Kong, only a mistake in the plans led to the Gosport Barracks being built in Hong Kong, and *vice versa*, and certainly they do not look English. The splendid barracks of the Royal Marines are beyond, and then come the huge earth forts that guard the land approach to Portsmouth. These forts are mostly garrisoned by Garrison Artillery, but Fort Brockhurst is the depôt for time-expired men of all cavalry and infantry regiments. "Time expired" means men who have finished the time abroad for which they enlisted, seven, eight, or twelve years, or, more rarely in these short service days, twenty-one years. There is a large time-expired depôt at Deohl-Allée, in India, where the men for Home are gathered from the different regiments ready for embarkation on a troopship. If their great-coats are not also time-expired they are replaced by old ones, and the men are all medically examined before leaving, to see if they need hospital treatment or care during the voyage Home. The sick are of course admitted to the ship's hospital or sick bay, and a certain number of orderlies of the Medical Staff Corps are employed during each trooping season in voyaging backwards and forwards, to attend the sick on board. The comforts on a troopship are limited—one tub of water per diem to twenty men, and half-a-pound of soap to a hundred men per diem. The decks, swabbed down every day, in some cases are never dry. Two blankets are served out to each man, and more often than not he has to sleep on these same damp decks. All soldiers, except those medically exempt, are obliged to remain barefoot on board a troopship, both going out and coming home, to avoid the decks being scratched with boots. What wonder that men from a tropical climate landing in a bitter English winter, after a comfortless voyage, constantly fall ill with pneumonia, bronchitis, &c.

The station hospital at Gosport is about a mile from the town, approached by a toll bridge crossing a creek. Behind and overshadowed by the large Haslar Naval Hospital, the unpretentious Military Hospital has to provide for the sick of a large garrison, including Fort Brockhurst with hundreds of "time-expired" men. Gosport Station Hospital is close to the sea wall where the blue waves lap gently in summer, and rage in fury through the storms of winter. The wards, built on the block system, are on the ground floor, and each contains twenty-five beds. Those time-expired men who are sick when the troopship comes in are generally brought straight from Portsmouth in an ambulance by the floating bridge across the harbour. Pneumonia of a virulent type is one of the most common complaints, and there are always many cases of nephritis, of rheumatism, and of congestion and abscess of the liver. Ague is particularly liable to attack the men; whilst with every batch there is some bad case of tubercle of lung—a soldier who just struggled on "to finish his seven or his twelve," but who has broken down on the voyage home. Many men do their best to hide their sickness, with a view to getting away to their homes and friends; and cases of pneumonia have been brought into hospital from the train, by which they would have got off, had they not fainted or become delirious, thus drawing attention to their condition. The longing for home of these poor fellows makes them wonderfully good patients, grateful for every little kindness. They are welcomed with their parrots and other pets, and made to feel that they have at last reached harbour. Some time-expired men, who have given their best years to the service of their country, never even see the harbour lights, and each year some lay down their arms for ever in the hospital. Others leave the ward as reserve men, with 6d. a-day for five years, and perhaps find their old homes broken up, their old associations spoiled, and thus, friendless indeed, have to begin to fight afresh the battle of life!

### Asylum News and Nursing.

[Contributions to this section should be addressed to The Editor, 428, Strand, W.C., and have the words "Asylum News" written in left-hand bottom corner of the envelope.]

### SECLUSION.

By seclusion is meant the enforced isolation of a patient, as when she is shut up in a room by herself. It does not matter whether the door be locked or no if the patient be not allowed to come out and mingle with the other occupants of the ward. A woman is as certainly secluded if a nurse be placed outside her room door to prevent her egress as if it be locked. The true test of seclusion is whether or no the patient be kept alone against her will. It seems a little absurd that she is considered to be secluded if locked up during the day but not if subjected to the same treatment at night. Seclusion in its proper place is a both efficacious and humane method of treating certain phases of insanity. It is unfortunate that it is so liable to be abused. The temptation to seclude a violent and refractory patient and thus escape from a trying and even dangerous responsibility is very great. At one time there is no doubt that seclusion was employed to an extent which was perfectly unjustifiable. It is questionable, however, whether the system of doing without it altogether can be defended with any more reason. We should be the last to advocate a return to its indiscriminate use. In those cases, however, in which it may be thought beneficial it should be used, even if the seclusion column of the medical journal be marred by an entry.

### JAMES MURRAY'S ROYAL ASYLUM, PERTH.

Though not so old as some of the others, yet prominent among asylum journals is *Excelsior*, the quarterly magazine of James Murray's Royal Asylum. It is extremely well got up, and in this respect bears off the palm among asylum magazines known to us. Some of the articles are very interesting, and we might more especially mention "Apud Helvetios et eorum finitimos" or "Notes of a Cheap Tour." These papers are throughout written in a bright and sparkling manner, the dispute in the railway carriage at the beginning of the journey being especially good. From the column given over to the "Wise and Otherwise" we select the following, which probably falls into the second category: "Why should a miss on being married become mistress, corrupted in spoken language to *missus*, and in written, abbreviated to Mrs.?" You can easily answer this, and I will as readily agree that you are right, and that the custom is justifiable. But why is there no distinction between bachelors and married men? You cannot answer this, and I therefore suggest a much-needed reform. Let bachelors retain the *Mister*, and let married men be introduced as *Got-her*.'

### A New Hospital for Crewe.

The Memorial Cottage Hospital at Crewe, to which Miss J. Rodgers has been appointed Matron, will be opened on July 10th. It contains beds for twenty-two patients, including a private ward. The hospital is built on one floor, the nurses' bed-rooms only being above. The little operating theatre, matron's and nurses' quarters, and the light, airy kitchens have been well planned, good cupboards and other conveniences being liberally provided.

### The Congress at Geneva.

The second International Gynæcological and Obstetric Congress will be held in Geneva in September, 1896. Professor Vulliet is anxious that the attention of readers of The Hospital should be called to the date, which, he says, has been erroneously announced for the present instead of the coming year.

### With St. Peter's Society at Meiringen.

II.

From Meiringen pilgrimages were made to many interesting places in the neighbourhood, or desultory wanderings in the woods round the hotel. We were blessed with glorious weather all the time, and the perfect freedom of action allowed to the members of our party was one of the chief charms of the trip.

Services were held in the hotel each Sunday, and were well attended by our own party and visitors from the other hotels in the place. A short distance away was the Swiss church. We used to watch the peasants on their way to worship. Some among them wore the quaint Swiss dress, but for the most part they were attired in ordinary English fashion. One Sunday four babies were brought to be christened. The little things were carried quite flat on boards, and seemed resigned, though uncomfortable. The name of each child was written on a slip of paper, and pinned to its chest—a bright idea for our English clergy—which would save many a nervous young godmother the ordeal of repeating the name many times.

One Sunday afternoon we joined a party for a walk up the Gorge of the Aare. A narrow frail-looking "gangway" runs for more than a mile along the face of the rock over the rushing waters of the river. This plank path—it is barely wider than a plank—is supported on slender iron girders. There is a handrail, but the whole effect is spidery and frail in the extreme. When we had traversed about half a mile of this path we became aware that we were followed by a band of gaily-dressed peasants armed with brass instruments. Without further prelude they began to tune up and played and sang several airs, winding up to our surprise with "God Save the Queen." Whether this was as a compliment to us or whether it was the German National Anthem, the tunes being the same, we could not find out.

There were many expeditions to Interlaken, both by road and rail. It is an eighteen-mile drive, passing Brienz, a lovely little place with eight miles of lake surrounded by snowy mountains. There is also at Brienz a railway and hotel, the latter most attractive (the Hotel de L'Ours). Interlaken is a town of shops and hotels, a perfect Brighton in the Alps. There is a superb view of the Jungfrau, and it is a centre for many beautiful walks; but the town is too much shut in and built over, and too civilised for those who wish only for cowbells and châlets.

A merry family party of sixty started for the Handeca Falls one morning in brakes, and some hay carts for those who did not mind jolting. We went up the steep mountain road, fresh glories breaking on us at every step. Here a picture of hanging woods, their blackness reflected in the silver lake beneath; then a turn in the road showing towering, to the sky an army of snowy peaks and jagged mountain tops. All along the road flowers grew, clinging to the rugged rocks and climbing up its face as flowers will; and above, the unutterable blue of the sky.

The horses were baited at Gutamen on loaves of bread cut up into small pieces which they devoured with much gusto, and after an hour's further drive Handeca was reached. The Falls proved every whit as grand as the guide book reported, and the rainbow was most beautiful. It stretched all along the rock and right across the lower part of the Fall, the effect on the water being lovely.

Many were the pilgrimages made in search of the Alpine rose. It grew round the Falls at Handeca and at Hohfluh. A walk leads to Hohfluh up a very steep but otherwise good driving road, with many little nooks and shady corners where the jutting rock gives shelter from the scorching sun. Of some Swiss girls inquiry for the flowers were made. They shook their heads, smiling, and pointed higher up the mountain. So, alas! the walk was in vain. The return to Meiringen was accomplished by train through Bruning, with several English for fellow passengers. All chatted like old friends. Oh! the wondrous sociability of the Englishman abroad compared with his supposed aversion to his fellow creatures in his own country. Then there is lovely Giersbach. Half-way across the lake to Interlaken, leaving the steamer, and ascending the sadly modern and up-to-date cable train, the terrace of the hotel is gained. The Falls are not so lovely, but perhaps more impressive than those at Handeck. One memorable walk is to Rosenlain, which seems the loveliest spot on earth, passing the Falls of Reichenbach, gazed at comfortably for the moderate charge of fifty centimes from the window of a little châlet opposite, and leaving them behind, higher and higher up to the glacier. A scene of marvellous beauty in its dazzling brightness. On the right, far below, lies the peaceful valley in the afternoon sun, and on the left, the bare and rugged bosom of the Engelhorn.

One afternoon was spent at Lurgern, reached by rail up the mountain side. The train panted and groaned up the hill, and finally came to a stop. There was a tedious wait of twenty minutes, presumably for the engine to recover itself! Space fails to tell of many other walks, and of many afternoons spent enjoying the beautiful view from the hotel garden.

The evenings were enlivened by competitions of floral bouquets and table decoration, arranged by the ladies of the party. Music was a great resource in that land of the jödel and the cowbell. One evening the Choral Union of the town gave a concert in the saal of the hotel. The voices were strong and pure and they sang with taste and precision in two parts only. The men and women sing separately, and do not seem to have any four-part music for all voices.

This short sketch of our fortnight in Switzerland may inspire some nurses with a desire to go upon the same delightfully economical terms as fell to the lot of the St. Peter's Society, of Walworth.

### Where to Go.

The Inner Temple Gardens are open to the public during June, July, and August, from six o'clock until dusk every evening except Saturday and Sunday.

Terry's Theatre.—A morning performance will be given on Thursday, July 13th, in aid of the Clarence Wing of St. Mary's Hospital. An excellent programme has been arranged.

Trained Nurses' Club, 12, Buckingham Street, Strand. —On Friday, June 14th, at a quarter to eight, Dr. Grigg will lecture on "The Most Frequent Complications of Child-bed: Their Recognition and Treatment." Tickets for non-members, 6d. each.

Hospital for Epilepsy and Paralysis, Regent's Park.—On May 24th, the in-patients and staff of nurses and servants enjoyed an excellent entertainment organised by Miss Ridley, the matron. Songs were ably rendered by Miss Mabel Buckler, Miss Adela Boora, Miss Florrie Orgill, and Mr. Stanley Raymond; and admirable recitations were given by Mr. H. J. Hunter and Mr. Herman-Prideaux Davson. Miss Nunn's charming violin solos, and a piano and banjo solo by Miss A. Lewns and Mrs. Stanley Raymond were enthusiastically received. Miss Osborne was chief pianist, a fine instrument having been kindly lent by Messrs. S. and P. Erard. Most of the acting physicians were present, and members of the committee and friends, including the hon. chaplain (the Rev. F. K. Povah), and a pleasant evening resulted.

